# Green Synthesis of Silver Nanoparticles Using Aerial Part Extract of the *Anthemis pseudocotula* Boiss. Plant and Their Biological Activity

**DOI:** 10.3390/molecules28010246

**Published:** 2022-12-28

**Authors:** Abdul-Wali Ajlouni, Eman H. Hamdan, Rasha Awwadh Eid Alshalawi, Mohammed Rafi Shaik, Mujeeb Khan, Mufsir Kuniyil, Abdulrahman Alwarthan, Mohammad Azam Ansari, Merajuddin Khan, Hamad Z. Alkhathlan, Jilani P. Shaik, Syed Farooq Adil

**Affiliations:** 1 Physics Department, College of Applied Sciences, Umm Al-Qura University (UQU), Makkah 21955, Saudi Arabia; 2 Quality Assurance Supervisor, Salehiya Medical Company, Riyadh 12242, Saudi Arabia; 3 Laboratory Specialist Poison Control and Forensic Chemistry Center in Riyadh, Ministry of Health, Riyadh 13211, Saudi Arabia; 4 Department of Chemistry, College of Science, King Saud University, P.O. Box 2455, Riyadh 11451, Saudi Arabia; 5 Department of Epidemic Research, Institute for Research & Medical Consultations (IRMC), Imam Abdulrahman Bin Faisal University, Dammam 31441, Saudi Arabia; 6 Department of Biochemistry, College of Science, King Saud University, Riyadh 11451, Saudi Arabia

**Keywords:** green synthesis, *Anthemis pseudocotula Boiss*, silver nanoparticles, antimicrobial, antibiofilm

## Abstract

Green syntheses of metallic nanoparticles using plant extracts as effective sources of reductants and stabilizers have attracted decent popularity due to their non-toxicity, environmental friendliness and rapid nature. The current study demonstrates the ecofriendly, facile and inexpensive synthesis of silver nanoparticles (AP-AgNPs) using the extract of aerial parts of the *Anthemis pseudocotula* Boiss. plant (AP). Herein, the aerial parts extract of AP performed a twin role of a reducing as well as a stabilizing agent. The green synthesized AP-AgNPs were characterized by several techniques such as XRD, UV-Vis, FT-IR, TEM, SEM and EDX. Furthermore, the antimicrobial and antibiofilm activity of as-prepared AP-AgNPs were examined by a standard two-fold microbroth dilution method and tissue culture plate methods, respectively, against several Gram-negative and Gram-positive bacterial strains and fungal species such as *Escherichia coli* (*E. coli*), *Staphylococcus aureus* (*S. aureus*), multidrug-resistant *Pseudomonas aeruginosa* (MDR-PA) and *Acinetobacter baumannii* (MDR-AB), methicillin-resistant *S. aureus* (MRSA) and *Candida albicans* (*C. albicans*) strains. The antimicrobial activity results clearly indicated that the Gram-negative bacteria MDR-PA was most affected by AgNPs as compared to other Gram-negative and Gram-positive bacteria and fungi *C. albicans*. Whereas, in the case of antibiofilm activity, it has been found that AgNPs at 0.039 mg/mL, inhibit biofilms formation of Gram-negative bacteria i.e., MDR-PA, *E. coli*, and MDR-AB by 78.98 ± 1.12, 65.77 ± 1.05 and 66.94 ± 1.35%, respectively. On the other hand, at the same dose (i.e., 0.039 mg/mL), AP-AgNPs inhibits biofilm formation of Gram-positive bacteria i.e., MRSA, *S. aureus* and fungi *C. albicans* by 67.81 ± 0.99, 54.61 ± 1.11 and 56.22 ± 1.06%, respectively. The present work indicates the efficiency of green synthesized AP-AgNPs as good antimicrobial and antibiofilm agents against selected bacterial and fungal species.

## 1. Introduction

Nanotechnology can offer solutions to numerous environmental and technical challenges in the areas of medicine, agriculture, food, electronics and wastewater treatment [1,2]. From the past few decades, there has been an augmented importance on the development of metal nanoparticles owing to their remarkable optical and electrical properties [3,4,5]. Excellent physico-chemical and physico-mechanical properties of metal nanoparticles have made them desirable aspirants for innovative applications in the medical field as antibiotic and anticancer agents [6,7]. In this context, noble metal nanoparticles such as platinum, copper, silver, gold, palladium, magnesium, zinc, and titanium, have attained significant consideration for medical applications due to their multifunctional capabilities [8,9,10]. However, physical approaches are preferred for the synthesis of high-quality metal nanoparticles [11], in the efforts to diminish toxic waste. Increasingly, considerable interests have been developed towards the advancement of novel wet-synthesis approaches which are efficient, inexpensive and can be easily perform by using eco-friendly reagents including natural products, etc. [12,13].

Natural products, termed as phytomolecules acquired from fungi, bacteria and plants have demonstrated excellent reducing potential during the preparation of metal nanoparticles due to substantial diversities in their chemical compositions [14,15,16]. Due to their reducing abilities, the active phytomolecules existing in the plant extracts not only facilitate the synthesis of nanomaterials but also assist in the stabilization of the surface of resulting nanoparticles during the synthesis [17,18,19,20]. Furthermore, it has also been confirmed by various studies that plant extracts containing bioactive secondary metabolites like tannins, terpenoids, polyphenols, alkaloids, amines, polysaccharides, ketones and aldehydes, flavonoids, lignin and proteins, etc., play the dual role as reducing and stabilizing agents in the conversion of metal ions into metal nanoparticles [21]. For example, *Origanum vulgare* L., *Pulicaria glutinosa*, *Cordia myxa*, etc. have been employed for the synthesis of a variety of nanoparticles, which have been selected based on their phytochemical composition [22,23]. Often, nanomaterials obtained by using plant extracts have been used for a variety of biomedical applications such as antimicrobial, anti-fungal agents, even as biosensors and for several other biological applications [24]. Recently, *Aegle marmelos* aqueous leaf extract was used to prepare Ag NPs which have demonstrated good antimicrobial activity against *Bacillus megaterium*, *Bacillus aryabhattai*, *Staphylococcus aureus*, *Serratia marcescens* and *Pseudomonas putida*. In this case, the highest zone formation was observed at (8.4  ±  0.3) in 100 µg/mL concentration of Am-AgNPs against *Serratia marcescens* [25]. Although silver nanoparticles have been extensively studied earlier, their synthesis with different media may produce a diverse quality of nanoparticles with different sizes, shapes, and compositions etc. These properties may have a significant effect on the resulting biological potential of the material. Therefore, it is beneficial to test the biological properties of NPs prepared with different sources of reagents. In this scenario, one such plant *A. pseudocotula* Boiss. has not been investigated for the synthesis of silver nanoparticles and an exploration of their biological potential. *A. pseudocotula* Boiss belongs to the family Asteraceae and is known to possess a variety of active phytochemicals such as sesquiterpene lactones, flavonoids and polyacetylenes [26].

**Scheme 1:**
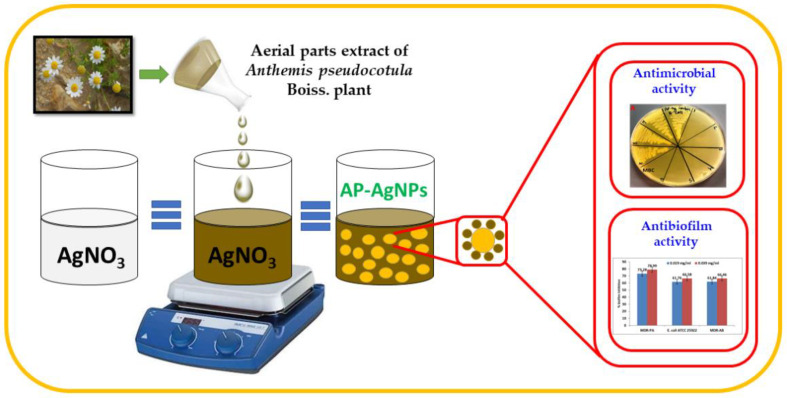
Graphical representation of silver nanoparticles (AP-AgNPs) synthesis using aerial part extracts of *A. pseudocotula* Boiss. plant.

Among different metallic nanoparticles, silver have been extensively utilized in various fields, including as an anti-microbial agent in different functional materials, as a potent coating agent, effective biosensors and as a catalyst in various chemical reactions. Furthermore, silver nanoparticles have also been known to possess effective biological properties such as anti-platelets and anti-HIV etc., [27,28,29,30,31]. Hence, in the present study, we report the synthesis of silver nanoparticles (AP-AgNPs) using the aerial part extract of *A. pseudocotula* Boiss. plant. The synthesized AP-AgNPs were characterized using several techniques such as XRD, UV-Vis, FT-IR, TEM, SEM, and EDX. In addition, during this study, antimicrobial and anti-biofilm properties of the as-prepared green synthesized AP-AgNPs were evaluated by using selected bacterial and fungal strains (Scheme 1).

## 2. Results and Discussions

### 2.1. UV-Vis Analysis

**Figure 1:**
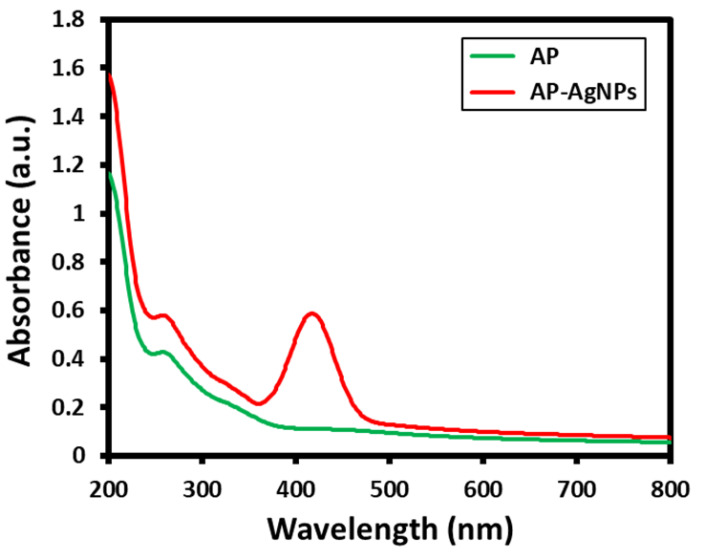
UV-Vis spectra of aerial parts extract of *A. pseudocotula* Boiss. plant (AP) and silver nanoparticles using aerial parts extract of *A. pseudocotula* Boiss. plant (AP-AgNPs).

The silver nanoparticles (AP-AgNPs) formation is monitored by a UV-Vis analysis which is carried out by collecting the reaction mixture at regular intervals; the formation of nanoparticles was confirmed by the color transformation in the reaction mixture from light green to dark brown. The UV-Vis analysis of the reaction mixture revealed that, in the beginning of reaction, the nucleation process progressed very rapidly, leading to the fast development of AP-AgNPs in the first hour of the reaction. After two hours, the nucleation process was slowed down and no further transformation was observed which is indicated by the unchanged intensity of the absorption band, this further confirmed the completion of reaction. The UV-Vis spectrum obtained a display of the absorption in the visible range from 370 to 460 nm, with a sharp intense peak that appeared at 424 nm (Figure 1) which confirms the formation of AP-AgNPs. Additionally, absorption peaks at 260 nm and 330 nm were also observed, which belongs to the phytomolecules from AP extract remaining on the surface of AP-AgNPs as capping agents, which is confirmed by the UV-Vis analysis of aerial part extract of *A. pseudocotula* Boiss. plant (AP). The surface plasmon resonance (SPR) of metallic NPs is typically sensitive to various factors, such as shape, size and inter-particles interactions (cluster formation) with the external medium [32]. This was indeed confirmed in our earlier study, in which an increase in the size of nanoparticles causes the shift of UV peak towards the longer wavelength from 427 (~40 nm size of NPs) to 459 nm (~60 nm size) [33]. This infers that the UV peak at a shorter wave-length (as appeared in this study at 424 nm) indicates towards the formation of smaller-size NPs.

### 2.2. XRD Analysis

**Figure 2:**
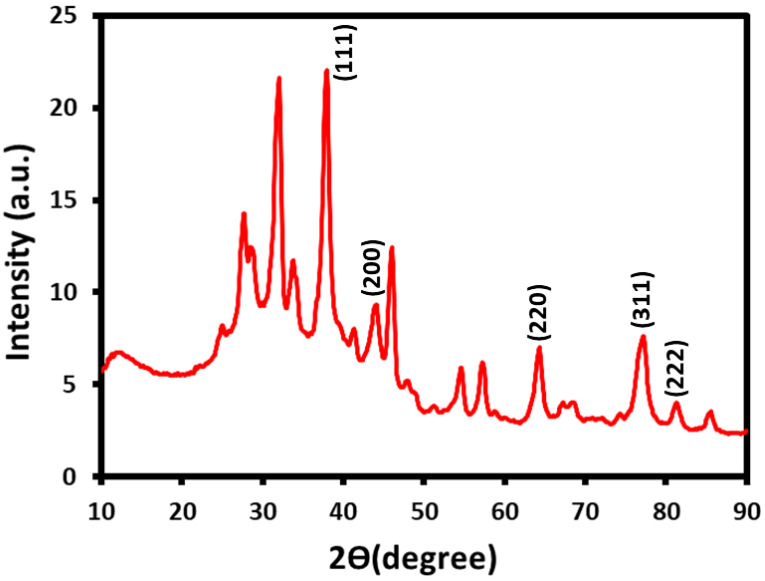
X-ray diffraction (XRD) diffractogram of as-synthesized silver nanoparticles (AP-AgNPs).

As-synthesized AP-AgNPs were characterized by an XRD analysis (Figure 2). The diffractogram pattern of AP-AgNPs exhibited some intense diffractions, which endorses the crystallinity of the as-synthesized AP-AgNPs. The five reflections, 37.75° (111), 44.41° (200), 64.23° (220), 76.95° (311) and 81.33° (222) clearly specified the development of the face-centered cubic (fcc) structure of the AP-AgNPs. Particularly, the nanocrystals growth direction is signified by the most intense reflection at 37.75° (111), additional reflections are also noticed apart from the reflections associated to AgNPs, which may be ascribed to inorganic residual moieties of the *A. pseudocotula* Boiss. plant extract on the surface of the AgNPs i.e., AP-AgNPs. Similar results were also obtained in case of silver NPs prepared by using *Salvadora persica* L. extract. It is noteworthy that the XRD peaks of AP-AgNPs are slightly shifted when compared to the standard silver XRD data (JCPDS no. 04-0783) published in the literature. For instance, the characteristic Ag peaks of AP-AgNPs appeared at 37.75°, 44.41°, 64.23°, 76.95° and 81.33°, whereas, the same peaks of standard silver appeared at 38.11°, 44.27°, 64.42°, 77.47° and 81.53°, respectively [34]. The insignificant difference in the peak positions can be associated to the presence of the residual Phytomolecules of *A. pseudocotula* Boiss extract on the surface of the as-obtained silver nanoparticles. The crystallites size of the powder of resultant Ag NPs were measured according to the Debye–Scherrer formula as given below.

(1) Debye–Scherrer formula D=kλβcosθ

(2) $$d\;spacing\;caluction\;d=\frac{a}{\sqrt{h2+k2+l2}}$$

where ‘*D*’ is the crystal size; ‘*d*’ is Interplanar Spacing; ‘*θ*’ is Bragg’s angle; *a* = Lattice Constant; *h*, *k*, *l* = Miller Indices; ‘*λ*’ is corresponding to the wave-length X-ray; and ‘*k*’ is the dimensionless shape factor; and ‘*β*’ is the line broadening at half the maximum intensity. The average crystallite size of the as-synthesized silver nanoparticles (AP-AgNPs) is found to be 9.16 ± 0.84 nm (Table 1).

### 2.3. FT-IR Analysis

**Figure 3:**
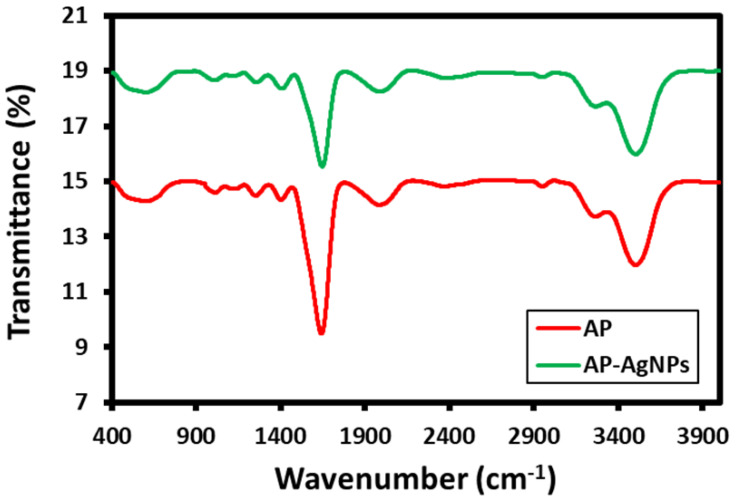
FT-IR analysis of aerial part extract of *A. pseudocotula* Boiss. plant (AP) and as-synthesized silver nanoparticles (AP-AgNPs).

The twin role of the aerial parts aqueous extract of *A. pseudocotula* Boiss. plant as a reductant and capping agent was established by relating the Fourier-transformed infrared spectra of aqueous extract of pure aerial parts of *A. pseudocotula* Boiss. plant and as-synthesized AP-AgNPs, as demonstrated in Figure 3. The FT-IR spectrum of AP-AgNPs is remarkably similar to the spectrum of pure aerial parts extract of *A. pseudocotula* Boiss. plant, except few marginal changes in some peaks. This striking similarity amid these two spectra AP-AgNPs and aerial part extract evidently recommends that some of the residual phytoconstituents of the aerial part aqueous extract stayed bonded on the surface of the AP-AgNPs. The FT-IR spectrum of aerial parts aqueous extract of *A. pseudocotula* Boiss. plant display peak at 3503 cm^−1^ corresponding to –OH group, and the peak at ~2930 cm^−1^ corresponds to −CH asymmetrical stretch from the phytomolecules. Furthermore, several peaks displayed in the FT-IR spectrum corresponding to numerous oxygen-comprising functional groups such as ~2130 cm^−1^ (C≡C), ~1654 cm^−1^ (C=O), 1392 cm^−1^ (C–O) and ~1000 cm^−1^ (C–O). The majority of these peaks are also existing in the FT-IR spectrum of AP-AgNPs with some marginal shifts. Consequently, the occurrence of these peaks in the FT-IR spectrum of AP-AgNPs is evidently directed towards the effective dual role of the aerial parts of aqueous extract of *A. pseudocotula* Boiss. plant, both as a reducing and stabilizing agent.

### 2.4. SEM and EDX Analysis

**Figure 4:**
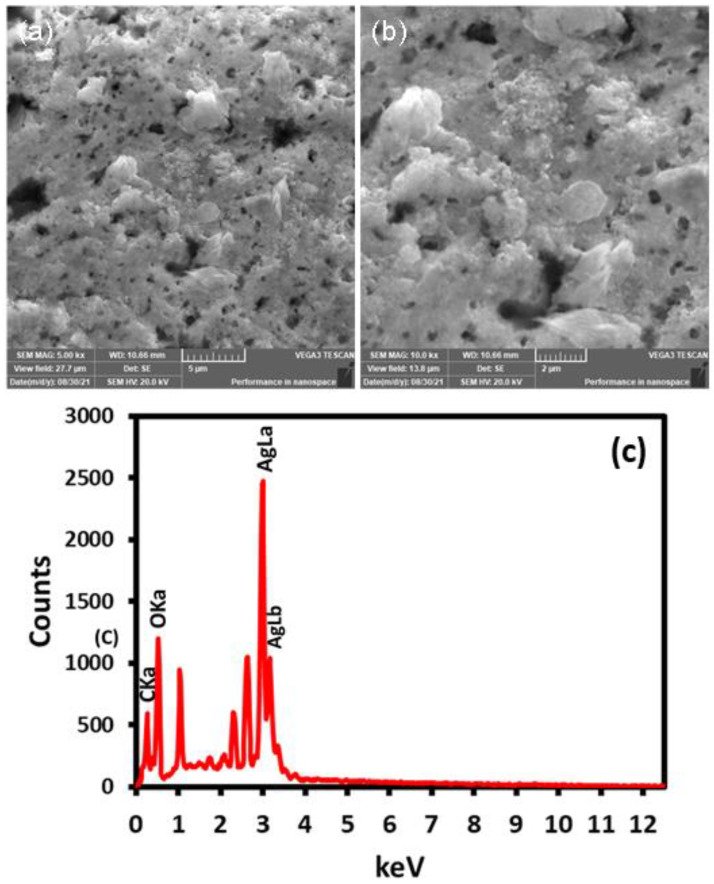
(**a**,**b**) scanning electron microscopy of the AP-AgNPs at two magnifications (5 and 10 kX) and (**c**) EDX analysis and elemental composition of the AP-AgNPs.

The scanning electron microscopic (SEM) analysis of as-synthesized silver nanoparticles (AP-AgNPs) is further carried out to investigate the surface morphology of the AP-AgNPs (Figure 4a,b). It is revealed that relatively spherical and uniform AP-AgNPs are formed with a diameter of 13 to 40 nm. The SEM image of as-synthesized AP-AgNPs obtained by using an aerial part aqueous extract of *A. pseudocotula* Boiss. plant is given in Figure 4a,b. The images suggest the presence of organic moieties on the surface of nanoparticles as stabilizing agents. The accumulation of phytomolecules possibly occurs due to the hydrogen bonding and/or electrostatic interactions between the functional groups of active phytomolecules and the surface of nanoparticles. Furthermore, the elemental composition analysis is confirmed by performing an EDX analysis (Figure 4c). The energy-dispersive X-ray (EDX) analysis revealed that the strong energy signals around 3–3.1 keV confirmed the existence of ‘Ag’, the main element, while other signals noticed in the range 0.0–2.6 keV, correspond to the presence of oxygen and carbon, indicating the occurrence of the residual organic compounds of the *A. pseudocotula* Boiss. plant aerial part extract on the surfaces of the AP-AgNPs. This further confirms the dual role of phytomolecules of the extract, i.e., both as a reducing as well as a capping agent.

### 2.5. TEM Analysis

**Figure 5:**
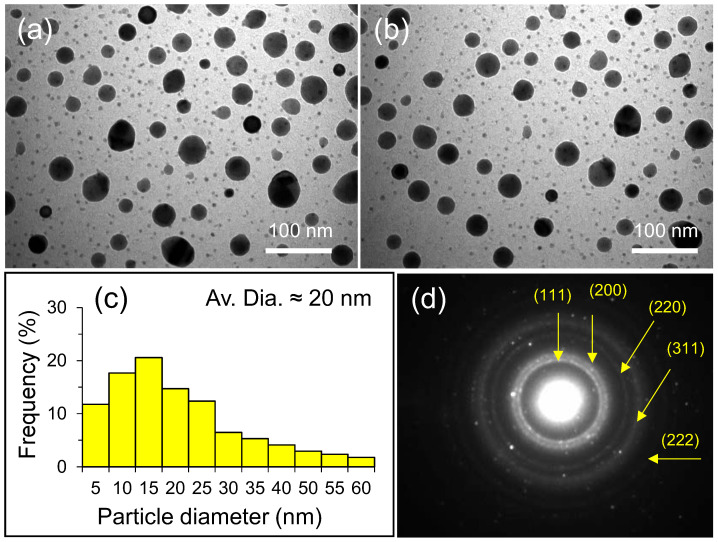
(**a**,**b**) TEM images, (**c**) size histogram and (**d**) SAED pattern of AP-AgNPs. The average size of the particles was measured around 20 nm. The characteristics rings of the AP-AgNPs are labelled in the SAED pattern.

The size and morphology of AP-AgNPs synthesized with aerial part aqueous extract of A. pseudocotula Boiss. plant is assessed by the transmission electron microscopic analysis (TEM). Figure 5a,b displays an overview of the AP-AgNPs, indicating the well-distributed nanoparticles with spherical morphology. Furthermore, the particle size distribution graph is estimated by using image J software (Figure 5c and the particle size distribution graph of the AP-AgNPs displays the average particle size is ~20 nm. The selected area electron diffraction (SAED) pattern depicts characteristics rings (Figure 5d), which designates that these AP-AgNPs are highly crystalline in nature, and the characteristic rings of the as-synthesized AP-AgNPs are labelled in the SAED pattern. The SAED pattern of AP-AgNPs also offers additional supportive information due to the appearance of five characteristic rings. These dark spotted rings points towards the lattice pattern of (111), (200), (220), (311) and (222) (Figure 5d). Indeed, the SAED pattern is nicely correlated with the aforementioned XRD data and offers clear proof of the conformation of the crystalline nature of resulting NPs.

### 2.6. Antimicrobial Activity of AP-AgNPs

**Figure 6:**
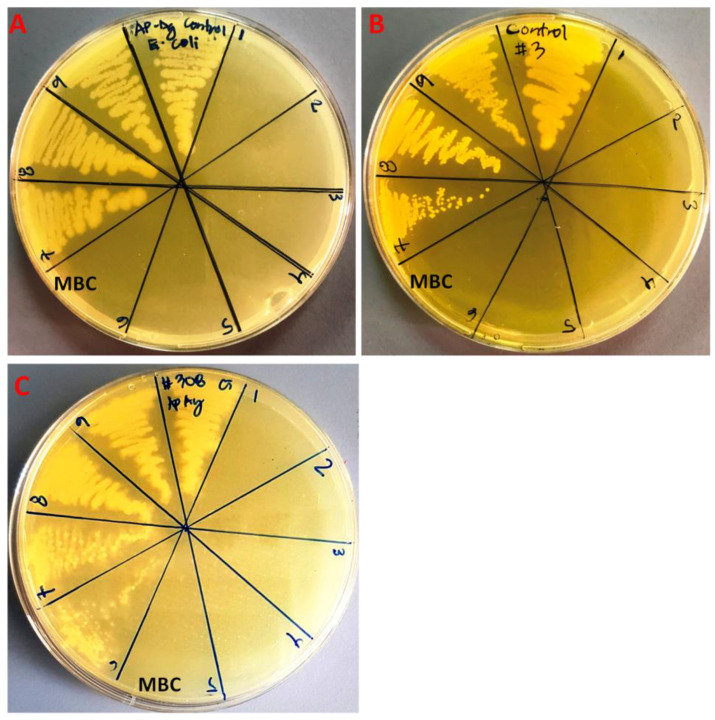
Nutrient agar plates showing MBC values of AP-AgNPs against Gram-negative bacteria. (**A**) *E. coli* (ATCC 25922), (**B**) multidrug-resistant *Pseudomonas aeruginosa*, and (**C**) multidrug-resistant *Acinetobacter baumannii*. Note: Block 1, 2, 3, 4, 5, 6, 7, 8 and 9 represents the concentration of AP-AgNPs i.e., 5, 2.5, 1.25, 0.625, 0.3125, 0.15625, 0.078125, 0.039 and 0.019 mg/mL, respectively, while 10 represents the control (untreated bacteria).

**Figure 7:**
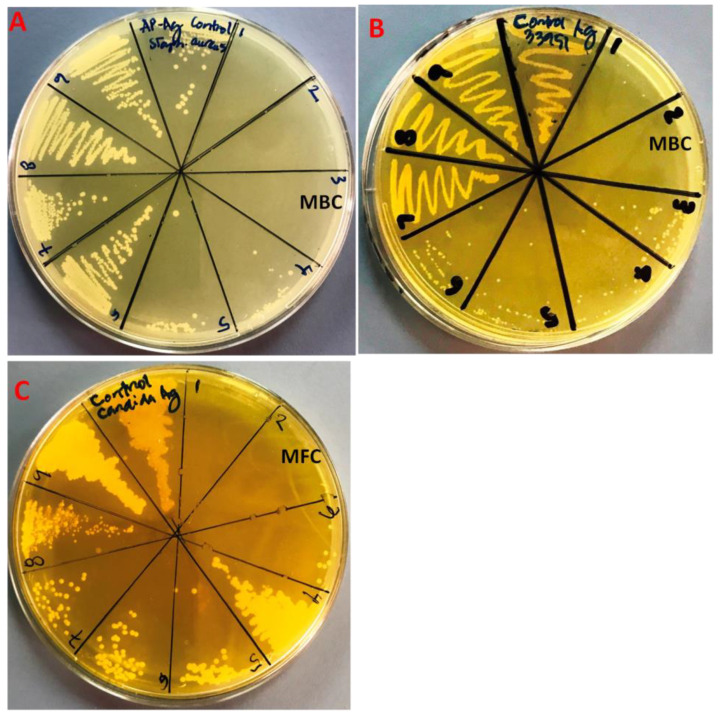
Agar plates showing MBC values of AP-AgNPs against Gram-positive bacteria (**A**,**B**) and fungi (**C**). (**A**) Represents *S. aureus* (ATCC 25923), (**B**) methicillin-resistant *S. aureus* and (**C**) represent fungi *C. albicans*. Note: Block 1, 2, 3, 4, 5, 6, 7, 8 and 9 represents the concentration of AP-AgNPs i.e., 5, 2.5, 1.25, 0.625, 0.3125, 0.15625, 0.078125, 0.039 and 0.019 mg/mL, respectively, while 10 represents the control (untreated bacteria and fungi).

The antimicrobial potential of as-biosynthesized AP-AgNPs (0.019 to 5 mg/mL) is investigated against the Gram-negative *E. coli*, MDR-*P. aeruginosa*, MDR- *A. baumannii*, Gram-positive *S. aureus* (ATCC 25923) and MRSA bacteria, and fungi *C. albicans* strain using microbroth dilution. The images in Figure 6 and Figure 7 clearly demonstrate the antimicrobial activity of AP-AgNPs against the tested organisms and drug strains. The MICs, MBCs and MFCs values of AgNPs against various strains were shown in Table 2. The MICs values of AP-AgNPs against Gram-negative bacteria i.e., MDR *P. aeruginosa*, *E. coli* (ATCC 25922) and MDR-*A. baumannii* were found to be 0.039, 0.078 and 0.078 mg/mL, respectively; whereas the MBC values were found to be 0.156, 0.156 and 0.312 mg/mL, respectively (Table 2; Figure 6). The MICs values of AP-AgNPs against Gram-positive bacteria i.e., *S. aureus* (ATCC 25923) and MRSA were 0.312 and 0.625 mg/mL, respectively, whereas the MBCs values were 1.25 and 2.5 mg/mL, respectively (Table 2; Figure 7A,B). However, The MIC and MFC value of AP-AgNPs against fungi C. albicans were 0.625 and 2.5 mg/mL, respectively (Table 2; Figure 7C). The MICs and MBCs/MFCs values of AgNPs in this investigation are in good agreement with earlier studies [35,36]. In a previous research, Ansari et al. [35]. showed that green-produced AgNPs had MIC values of 258–758 µg/mL and MBC/MFC values of 516–1533 µg/mL, respectively, against MDR-PA, MRSA and C. albicans. Whereas, in another research, it has been reported that biogenic AgNPs demonstrated MIC and MBC values of 39–78 µg/mL and 156–312 µg/mL against MDR-P. aeruginosa and MRSA, respectively [36]. The results have revealed that the biosynthesized AP-AgNPs showed a maximum antimicrobial activity against Gram-negative bacterial strains when compare to the Gram-positive bacteria, while weak antifungal activity was observed against the studied fungal strain (Table 2). Among Gram-negative bacterial strains, MDR-P. aeruginosa clearly stands out as the most effected bacteria by AP-AgNPs. The difference in antibacterial activities of both Gram-negative and Gram-positive bacterial strains were mainly attributed to the structural difference in their respective cell walls [37]. The cell wall of Gram-negative bacteria is thinner than that of Gram-positive bacteria and therefore does not give as much protection to the cells [38]. The peptidoglycan layers of Gram-negative cell wall are relatively thinner (7 to 8 nm) than their counterpart, which can be damaged easily. The major component of Gram-negative bacteria is lipopolysaccharide, which is highly negatively charged, thus interacting with positively charged AP-AgNPs more quickly and are hence more susceptible to AP-AgNPs. On the other hand, the cell wall of Gram-positive bacteria has a very thick cell wall, mainly composed of rigid peptidoglycans layers (80%) that provides additional rigidity and extraordinary support to resist against nanoparticles [35]. Still, there are contradictory claims regarding the antimicrobial properties of AgNPs against both strains, and some studies have also indicated the strong antibacterial properties of AgNPs against Gram-positive bacterial strains. Nevertheless, the majority of the studies suggest that the Gram-negative bacterial strains are more sensitive to AgNPs in comparison with Gram-positive bacterial strains. Reportedly, the antibacterial properties of phytomolecules-mediated AgNPs occurs with several mechanisms, including ROS (reactive oxygen species) generation, un-wanted interactions of AgNPs with the DNA of bacterial cell walls and the release of silver ions from the AgNPs [39]. For instance, Pal et al. have proposed silver ion-led antimicrobial properties of differently shaped nanoparticles. In this case, ions of silver nanoparticles interacted with the bacterial cell wall which inhibited a respiratory enzyme(s), facilitating the generation of reactive oxygen species and consequently damaging the cell [40].

### 2.7. Effects of AP-AgNPs on Biofilm Formation

**Figure 8:**
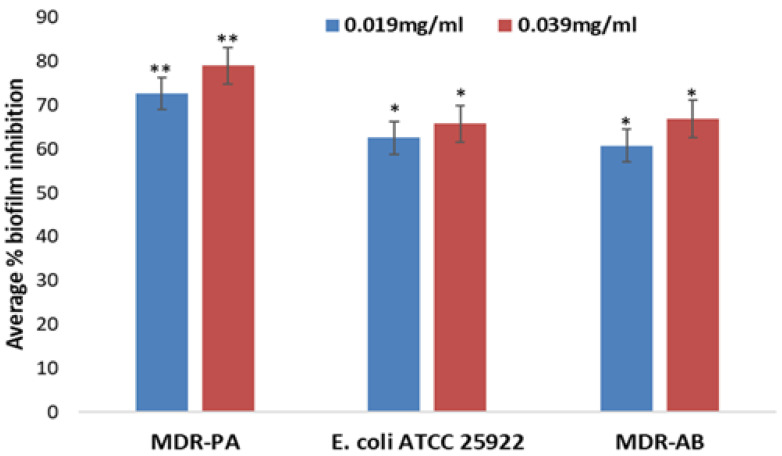
Inhibition of biofilm formation of MDR-PA, *E. coli*, and MDR-AB caused by AP-AgNPs treatment. The asterisks (**) represent the significance as *p* < 0.001 and asterisks (*) represent the significance as *p* < 0.01 against their control experiments by One-Way ANOVA-based Pairwise Multiple Comparison Procedures (Holm-Sidak method), whereas the overall significance level was 0.05.

**Figure 9:**
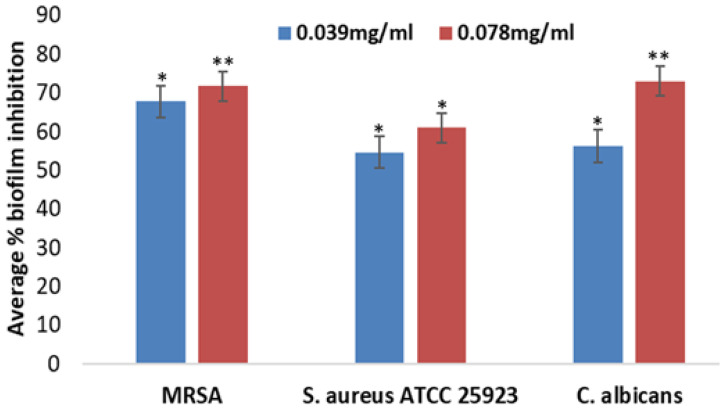
Inhibition of biofilm formation of MRSA, *S. aureus*, and *C. albicans* caused by AP-AgNPs treatment. The asterisks (**) represent the significance as *p* < 0.001 and asterisks (*) represent the significance as *p* < 0.01 against their control experiments by One-Way ANOVA-based Pairwise Multiple Comparison Procedures (Holm-Sidak method), whereas the overall significance level was 0.05.

The colonization of planktonic microbial cells such as bacteria and fungi result in the formation of multispecies biofilms, which renders a broad spectrum of antibiotics ineffective. A wide variety of metallic NPs have been considered as a possible alternate method to get around persistent biofilm diseases [41]. In the present study, the effects of sub-MIC i.e., (MIC/2) values of as-prepared AP-AgNPs on biofilm-forming abilities of Gram-negative bacteria, i.e., MDR-PA, E. coli ATCC 25922, MDR-AB and Gram-positive bacteria i.e., MRSA, *S. aureus* ATCC 25923 and fungi *C. albicans* was examined by 96-well polystyrene crystal violet assays. Figure 8 and Figure 9 exhibit that AP-AgNPs inhibit the biofilms formation of tested bacteria and fungi in a dose-dependent manner. It has been found that AP-AgNPs at 0.039 mg/mL inhibit the biofilms formation of Gram-negative bacteria i.e., MDR-PA, *E. coli* ATCC 25922 and MDR-AB by 78.98 ± 1.12, 66.14 ± 1.05 and 66.94 ± 1.35%, respectively (Figure 8). On the other hand, at the same dose (i.e., 0.039 mg/mL), AP-AgNPs inhibits the biofilm formation of Gram-positive bacteria i.e., MRSA, *S. aureus* ATCC 25923 and fungi *C. albicans* by 67.76 ± 0.99, 54.61 ± 1.11 and 56.22 ± 1.06%, respectively (Figure 9). The biofilm inhibition results revealed that AP-AgNPs inhibit the biofilm formation of Gram-negative bacteria more strongly than that of Gram-positive bacteria and fungi *C. albicans*. Overall, the obtained trends in biofilm formation suggest that the biosynthesized AP-AgNPs had great potential to inhibit the biofilm formation of several human pathogens such as drug-susceptible and drug-resistant Gram-positive and Gram-negative bacteria and fungi i.e., *C. albicans*. Previous investigations have revealed that AgNPs synthesized by *Terminalia catappa* leaf extract exhibit the similar pattern of anti-biofilm activity and it was found that AgNPs significantly inhibits the biofilm formation of MDR-PA, MRSA and *C. albicans* by 73.7, 69.56 and 63.63%, respectively, at a concentration of 7.8 µg/mL [41]. Allemailem et al. (2022) reported that AgNPs synthesized by ajwa date seed extract inhibits biofilm formation in *E. coli*, *S. aureus*, *P. aeruginosa*, *Enterococcus faecalis and Klebsiella pneumonia* by 70, 66, 39, 43, and 41%, respectively, at 50 µg/mL of AgNPs [42]. In another study, Ansari et al. (2021) found that 100 µM AgNPs reduced the biofilm by 54 to 85% in Gram-positive and Gram-negative bacteria and *C. albicans* at 500 µg/mL of AgNPs synthesized by polyherbal drug LIV52 [35].

## 3. Materials and Methods

### 3.1. Materials

The *A. pseudocotula* Boiss. is abundantly available in the Riyadh region and was collected from Northern Riyadh, Saudi Arabia. After the collection of plant material, the fresh plant was firstly identified by a plant taxonomist at King Saud University. AgNO_3_ (99.8%, Sigma Aldrich, St. Louis, MO, USA) and other chemicals and solvents applied in this work were purchased from Sigma Aldrich.

### 3.2. Methods

#### 3.2.1. Preparation of Aerial Part Extract of *A. pseudocotula* Boiss. Plant

To start with, fresh aerial parts of *A. pseudocotula* Boiss. plant was first dried at room temperature and grinded into small pieces. About 1.0 Kg of *A. pseudocotula* aerial parts were refluxed for ~3 h on a heating mantle at 80 °C. After three hours of being refluxed, the solution was allowed to cool down and be filtered. The water extract obtained was dried completely on a rotary evaporator to have a dark-brownish color extract. The resultant extract was stored at room temperature and was used for the synthesis of AgNPs. A total of 1.0 g extract from aerial parts of *A. pseudocotula* was dissolved in 10 mL of DI water and was used for the NPs synthesis.

#### 3.2.2. Green Synthesis of Silver Nanoparticles Using Aerial Part Extract of *A. pseudocotula* Boiss. Plant (AP-AgNPs)

The synthesis of silver nanoparticles (AP-AgNPs) was carried out by adding 1 mL from the stock solution of aerial part extract of *A. pseudocotula* Boiss. plant to 50 mL of 0.5 mM of AgNO_3_ aqueous solution in a 250 mL flask. The flask with resultant solution is attached with a magnetic stir bar and fixed on a condenser. The reaction was continued for ~120 min at 90 ± 5 °C. While stirring, the reaction color transformed from a light green to a brown color and afterwards no further color transformation is perceived until the end of the reaction. In order to eliminate any prospect of the existence of unbound residual phytomolecules on the surface of the green as-synthesized AP-AgNPs, it is re-dispersed in double distilled water via sonication for 20 min after centrifugation for 20 min. This procedure is continued three times to isolate pure AP-AgNPs. Afterwards, a reaction product is washed three times with double distilled water, and then the remaining solid was dried at 70 °C for 12 h in an oven. Finally, a black powder is achieved.

### 3.3. Characterization

A UV-Visible analysis was performed on lambda 35, Perkin Elmer, Waltham, MA, USA. A Fourier transform infrared analysis were performed on a 1000 FTIR instrument, Perkin-Elmer Waltham, MA, USA. The X-ray powder diffraction analysis of the as-prepared silver nanoparticles was performed on D2-Phaser, Bruker, Germany. Transmission electron microscopy (TEM) analysis was performed using JEM-1101, Jeol, Japan. Scanning electron microscopy (SEM) and an energy-dispersive X-ray analysis was performed using JSM 7600F instrument, JEOL, Tokyo, Japan.

### 3.4. Strains and Media

*S. aureus* (ATCC 25923), *E. coli* (ATCC 25922), multidrug-resistant *P. aeruginosa* (MDR-PA), methicillin-resistant *S. aureus* (MRSA, ATCC 33593), multidrug-resistant *A. baumannii* (MDR-AB) and *C. albicans* (ATCC 14053) strains were used for antimicrobial and antibiofilm activity assessment of the as-synthesized silver nanoparticles (AP-AgNPs). The clinical isolates i.e., MDR-PA and MDR-AB, were obtained from the Department of Epidemic Disease Research, Institute of Research and Medical Consultations, Imam Abdulrahman Bin Faisal University, Dammam, Saudi Arabia. The bacterial and *Candida* cultures were maintained in Luria Bertani (LB) and Sabouraud dextrose (SD) broth, respectively.

### 3.5. Assessment of Antimicrobial Efficacy of AgNPs

#### 3.5.1. Minimal Inhibitory Concentration (MIC), Minimal Bactericidal Concentration (MBC) and Minimal Fungicidal Concentration (MFC) Determination

The antibacterial and antifungal properties of as-prepared AgNPs is evaluated by using a microbroth dilution method in the range of 0.019 to 5 mg/mL against three Gram-negative bacterial strains (i.e., *E. coli* ATCC 25922, multidrug-resistant *P. aeruginosa* and multidrug-resistant *A. baumannii*), two Gram-positive bacterial strains (i.e., *S. aureus* ATCC 25923 and *methicillin resistant S. aureus*) and one medically important fungal strain (i.e., *C. albicans*). The tests were performed according to the procedure described by Elsharif et al. [43]. The MIC is defined as the lowest concentration of AP-AgNPs at which no visible growth is seen. After the MIC assessment, aliquots of 100 µL from wells (with no visible growth) were further spread on MHA and SDA plates for 24 h at 37 °C and 28 °C, respectively, to examine the MBC and MFC values of AP-AgNPs. The concentration of AP-AgNPs at which no colonies of bacteria and fungi was grown is considered as MBC/MFC values [44].

#### 3.5.2. Inhibition of Biofilm Formation by AgNPs

Further, the antibiofilm potency of AgNPs against bacteria and fungi is assessed using 96-well crystal violet microtiter assays with slight modification [45]. In brief, a fresh 20 µL of microbial suspension of *S. aureus* ATCC 25923, *E. coli* ATCC 25922, *methicillin resistant S. aureus* (MRSA), multidrug-resistant *P. aeruginosa* (MDR-PA), multidrug-resistant *A. baumannii* (MDR-AB) and *C. albicans* is inoculated with sub-MIC values of AP-AgNPs in 180 µL of culture media for 24 h at 35 °C. The cells without AP-AgNPs are taken as a control. After 24 h of incubation, the content of each well was removed from the 96-well plates and then washed twice with PBS to further remove the loosely attached cells. The adhered biofilm was stained with 0.1% crystal violet and washed again with PBS. The stained biofilm was solubilized with ethyl alcohol (95%) and the optical density was estimated at 595 nm and then the percentage of biofilm inhibition was estimated using the equation given below [46].

% Biofilm inhibition = [(OD of control − OD of tested samples)/(OD of control)] × 100(3)

### 3.6. Statistical Analysis

For a statistical analysis, each experiment was performed in triplicates, and the findings were reported as means ± standard deviation (SD). Onaway ANOVA was used to assess the significance levels of results at *p* < 0.05. One-way ANOVA-based Pairwise Multiple Comparison Procedures (Holm-Sidak method) was conducted on Sigma Plot 11.05 statistical analysis software.

## 4. Conclusions

This present study demonstrates the green synthesis of AP-AgNPs by using aqueous aerial part aqueous extract of *A. pseudocotula* Boiss. Materials based on silver nanoparticles (silver NPs) are among the most valuable functional materials. They are renowned for their distinctive properties, including their significant biological potential, which has made them vital components in many consumer items. The as-synthesized AP-AgNPs (20 nm) are characterized by various characterization techniques such as UV-Vis, XRD, FT-IR, SEM, EDX and TEM. The antimicrobial and antibiofilm activity of AP-AgNPs were examined against *E. coli*, *S. aureus*, methicillin-resistant *S. aureus*, multidrug-resistant *P. aeruginosa*, multidrug-resistant *A. baumannii* and *C. albicans* demonstrated that AgNPs exhibits maximum antimicrobial activity against Gram-negative bacterial strains followed by Gram-positive bacteria and fungi. Additionally, the biofilm inhibition results revealed that AP-AgNPs inhibit the biofilm formation of Gram-negative bacteria more strongly than that of Gram-positive bacteria and fungi *C. albicans*. As a result, the plant extract from *A. pseudocotula* Boiss. has a tremendous potential for creating different metallic nanoparticles as well as other useful nanomaterials. The synthesized AP-AgNPs can also be used as a potential candidate for antibacterial and antibiofilm agents.

## Figures and Tables

**Table 1:** XRD crystallographic data of the AP-AgNPs.

2θ (°)	d Space (nm)	(h, k, l)	a (nm)	Crystallite Size, D (nm)
37.9	0.24	111	0.41	7.96
46.0	0.20	200		11.64
64.2	0.14	220		8.68
77.0	0.12	311		7.01
81.3	0.12	222		10.52

**Table 2:** MIC, MBC and MFC (mg/mL) values of AP-AgNPs tested against various strains.

Strains	MIC (mg/mL)	MBC (mg/mL)
*MDR-P. aeruginosa*	0.039	0.156
*E. coli* ATCC 25922	0.078	0.156
*MDR-A. baumannii*	0.078	0.312
MRSA ATCC 33593	0.625	2.5
*S. aureus* ATCC 22923	0.312	1.25
*C. albicans ATCC 14053*	0.625	2.5

## Data Availability

Data are contained within the article.
